# Relationship between age, refractive errors and motor fusion in a normal Chinese adult population: a cross-sectional study

**DOI:** 10.1186/s12886-021-02105-z

**Published:** 2021-09-23

**Authors:** Man She, Tao Li, Qianqian Hu, Jie Zhu, Xiaodong Zhou

**Affiliations:** Department of Ophthalmology, Jinshan Hospital of Fudan University, 201508 Shanghai, China

**Keywords:** refractive errors, motor fusion, age, synoptophore

## Abstract

**Background:**

To investigate the relationships between motor fusion and sex, age and spherical equivalent (SE).

**Methods:**

This observational study enrolled 243 healthy, nonstrabismic adults, including 94 men and 149 women aged 20 to 59 years. The subjects were divided into three groups according to SE: myopic, emmetropic and hyperopic groups. The subjects were also divided into four groups according to age: 20–29, 30–39, 40–49 and 50–59 years groups. Motor fusion was measured with a synoptophore, including subjective angle (SA), divergence, convergence and fusional vergence range (FVR).

**Results:**

The mean values of divergence, convergence and FVR for the whole sample group were 9.72 ± 0.26°, 19.34 ± 0.54°, and 29.06 ± 0.62°, respectively. A higher value of divergence was found in the myopic group than in the emmetropic group (*p* < 0.05). SE and divergence were significantly different among age groups (all *p* < 0.05). In addition, linear regression analysis showed that SE was correlated with divergence (*p* = 0.003). Age was correlated with SE, divergence and FVR (*p* < 0.001, *p* = 0.005, *p* = 0.002, respectively). In addition, the proportion of SA being in the comfort zone (defined as the value of SA satisfying Percival’s criterion) in the age groups was significantly different (χ^2^ = 8.283, *p* = 0.041).

**Conclusions:**

Motor fusion is associated with age and SE in the normal Chinese adult population.

## Background

Binocular single vision (BSV) is a brain process in which the integration of two retinal images into a single visual perception is achieved [1]. It is crucial for one’s perception in both static and dynamic situations using BSV [2]. Normal BSV requires accurate eye alignments and binocular mechanisms for sensory fusion, vergence function and stereopsis [3], which can be measured by synoptophore. Worth [4] classified binocular function into three levels: superposition, fusion and stereovision. Superposition, also named simultaneous perception, is the ability of both eyes to sense the objects at the same time. On synoptophore, it means the coverage of one examination slide (e.g., car) with the second (e.g., door), which is also recorded as subjective angle (SA) [5]. It reflects a baseline estimate of habitual binocular vergence position [6]. Fusion is the ability to connect sense from one eye with sense from the other eye, with the necessity of both correct sensor and motor predispositions [7]. A synoptophore can usually be used to examine sensory and motor fusions. To examine sensory fusion, two slides with images that are flawed but complement each other are used [1]. For example, in one slide, there is a cat without a tail but a butterfly, whereas in the other slide, the same cat has a tail without the butterfly. If there was a match, the patient sees the cat with all features (tail and butterfly). Otherwise, there is no matching. Motor fusion is the ability to maintain binocular vision through a range of induced vergences until binocular vision is interrupted and the patient experiences diplopia [8]. The amplitude of motor fusion, including convergence and divergence, is usually measured with the synoptophore set at the position of SA. Stereovision refers to the ability of the eyes to have three dimensions (3D) of spatial perception.

Previous studies have found that BSV function may be associated with refractive characteristics and different ages. In 1995, Dwyer and Wick [9] found that a higher proportion of accommodative or binocular abnormalities was associated with some form of refractive errors. It was reported that convergence and divergence insufficiencies were associated with refractive error groupings. Convergence insufficiency was more likely to occur in patients with a lower degree of myopia, while divergence insufficiency was more likely to occur in patients with a higher degree of myopia [10]. Nevertheless, previous studies on BSV primarily focused on patients with significant amount of heterophoria, amblyopia or strabismus but not a normal binocular population. The relationship of motor fusion with refractive errors as well as demographic variables (gender, age and so on) in the normal population will help provide information on a subject’s binocular status and ability to compensate for the natural resting position of their eyes.

The amplitude of motor fusion reflects one aspect of binocular control, and a reduction can result in symptoms. Previous studies have found that convergence insufficiency could lead to asthenopia and visual discomfort [11–13]. O’Connor [14]reported that sensory and motor fusion are beneficial in the performance of motor skills tasks. Fusion amplitude measured by synoptophore and incidence of insufficient convergence were found to be significantly worse in the student group than in the non-student group [15]. To obtain a good understanding of the amplitude of motor fusion, the zone of clear single binocular vision (ZCSBV) is of great importance. It is the set of vergence and focal stimuli that the patient can see clearly while maintaining binocular fusion [16]. To measure it, the examiner finds the maximum convergence and divergence for which the patient sees a well-focused and single target. Percival [17, 18] stated that the comfortable subregion is the middle third of the ZCSBV, and he proposed that the demand line or eye alignment position should lie inside this subregion; otherwise, the patient will experience discomfort in binocular vision. Percival’s criterion is usually used to instruct prescriptions of prism or spherical lenses, but whether the fulfilment of Percival’s criterion is related to age or refractive errors in normal adult subjects remains unclear.

The purpose of this study was to investigate binocular parameters, including SA, divergence, convergence and fusional vergence range (FVR), in a healthy adult population and to evaluate the relationship between motor fusion and age, sex and refractive status.

## Methods

### Subjects

This was a single-centre, cross-sectional study conducted from September 1, 2018, to November 30, 2019, at Jinshan Hospital of Fudan University. A total of 243 clinical subjects were enrolled in this study, including 94 male and 149 female subjects aged 20 to 59 years. All the subjects were divided into 4 groups according to their ages (20–29; 30–39; 40–49; 50–59 years groups). The inclusion criteria were as follows: (1) normal general and ocular health (no known physical deficit or ophthalmic defect); (2) the visual acuity (VA) of at least 6/60 for each eye and the best corrected visual acuity (BCVA) for each eye should be 6/6 or better; (3) right position of eyes (with normal ocular alignment in the Hirschberg corneal reflex test) and normal eye movements; (4) absence of heterotropia on the cover test; and (5) absence of an ophthalmoscopically detectable organic lesion; (6) having normal stereoscopic vision. The exclusion criteria were strabismus or strabismic surgery, microtropia or heterophoria (1 exophoria with a standard deviation of ± 2 PD at distance and 3 exophoria with a standard deviation of ± 3 PD at near), diplopia, medication with ocular side effects, or neurological and circulatory illness.

The study was approved by the Ethical Committee of Jinshan Hospital of Fudan University(IEC-2020-S41). This study complies with The Code of Ethics of the World Medical Association (Declaration of Helsinki) for experiments involving humans and uniform requirements for manuscripts submitted to biomedical journals. Written informed consent was obtained from all the subjects.

### Examination

All participants underwent the clinical examination, including eye position, eye movement, uncorrected distant VA, cover tests, stereopsis examination, slip lamp examination, fundus examination, subjective refraction, and synoptophore tests.

1. The cover test for heterotropia and the cover-uncover test for heterophoria were performed. Anterior segments of the eyes were examined by slit lamp for the condition of corneal or lens, and ophthalmoscopic examination was performed for the presence of any organic lesions of the posterior pole of the fundus and the optic nerve.
2. Distant stereopsis was examined using qualitative analysis of stereoscopic pictures, with the results divided into existent or non-existent distant stereopsis[5]. Then, near stereoacuity was tested using the Titmus stereotest. Enough resting time between the measurement of distant stereopsis and near stereopsis was given to the participants to avoid fatigue and its negative effects on the test results. Participants who could identify the contents of the maximal arcsec picture were recorded as existent of near stereopsis.
3. Subjective refraction was measured after an autorefractor (RK-F1; Canon Corporation, Tokyo, Japan). Measurements were taken under noncycloplegic conditions. The tests were performed by an experienced optometrist. The spherical equivalent (SE) was calculated as a sphere plus half of the negative cylinder. Participants were then grouped into three refractive error categories based on SE according to the following criteria: (1) emmetropia, SE of both eyes were within ± 0.5D; (2) hyperopia, either both eyes higher than + 0.50 D or only one eye was higher than + 0.50 D, and the fellow eye was emmetropic; (3) myopia, myopic degrees of both eyes higher than − 0.50 D or only one eye was higher than − 0.50 D and the fellow eye was emmetropic [19]. As in the condition that one eye was myopic and the fellow eye was hyperopic, they were not included in the analysis [11].
4. Motor fusions were measured with a synoptophore (TSJ-IV, type A, Changchun Photoelectric Instrument Co., Ltd. China). All patients with refractive errors were told to wear the glasses that provided BCVA for 1–2 weeks before attending the synoptophore exam. Synoptophore tests were performed according to Ohyagi’s study [20]. Different targets using slides, one of a car and the other of a door, were presented for each eye through separated mirror tubes with an installed convex lens (+ 6.5D). The two tubes of the synoptophore were welded to allow movements horizontally. The subjects were requested to fuse the targets when seeing each slide separately according to the corresponding eye. The value of SA was then obtained when the targets were fused when a car was seen in the middle of the door. Then, motor fusion was measured with the synoptophore set at the position of SA using slides with a butterfly and cat. While the synoptophore tubes were slowly abducted or adducted, the subjects had to diverge or converge to maintain the fusion of the targets for as long as possible. When the subjects signed that a disruption of fusion appeared (the subjects saw two cats), divergence and convergence were measured using this procedure. Then, the FVR was calculated as the value of convergence plus the value of divergence. Normal SA in the comfort zone was defined as the value of SA satisfying Percival’s criterion.

### Statistical analysis

Both eyes of each subject were examined, and the right eyes were used for analysis. IBM SPSS V.22.0 software was used for data analysis. Given that the data did not fully comply with a normal distribution, the Mann-Whitney test was used to detect differences between sexes, while the Kruskal-Wallis test was used to detect differences among different age groups and refractive groups. Linear regression analysis was performed to assess the relationship between age, SE and divergence, convergence and FVR. Proportions of normal SA were compared using the chi-square test. All p values were two-sided and considered statistically significant when *p* < 0.05.

## Results

There were 243 subjects (94 males and 149 females) aged 20–59 years enrolled in this study. For the whole sample group, the average age was 42.1 ± 0.61 years old, and the average value of SE was − 0.99 ± 0.10. The mean values of divergence, convergence, and FVR were 9.72 ± 0.26°, 19.34 ± 0.54° and 29.06 ± 0.62°, respectively. The demographic characteristics and visual characteristics of the samples are shown in Table 1. The median ages for males and females were 42 and 44 years, respectively. The median values and 1st quartile and 3rd quartile of SE, divergence, convergence and FVR are shown in Table 1. No significant differences in these values were observed between sexes (all *p* > 0.05).

**Table 1:** Demographic and visual characteristics according to gender

	Male(*n* = 94)	Female (*n* = 149)	Z	P value
**Age (years)**	42 (31, 50)	44 (34,50)	-1.516	0.13
**SE (D)**	-0.5 (-1.75, 0)	-0.5(-1.25,0)	-1.264	0.21
**Divergence (°)**	9 (6, 12)	10 (7, 12)	-0.479	0.63
**Convergence (°)**	20 (14, 15.25)	18 (13.5, 23)	-1.314	0.19
**FVR (°)**	29 (22, 36)	28 (22, 34)	-0.666	0.51

**Fig. 1:**
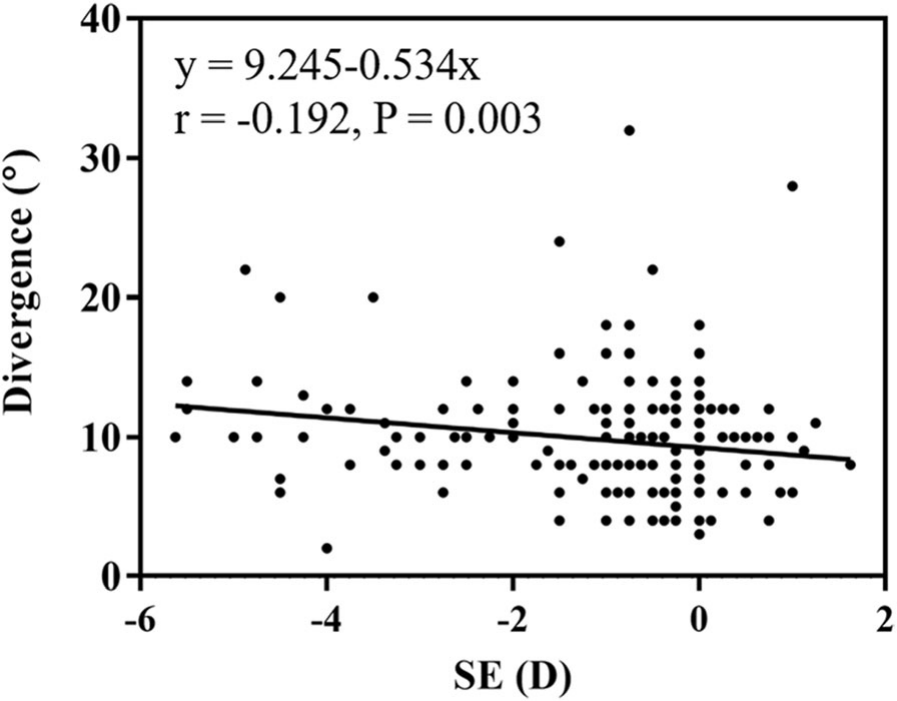
The relationship between SE and divergence. The mean age was 42.1 ± 0.61 years, ranging from 20–59 years. SE, spherical equivalent

As shown in Table 2, the median age was 54 years for hyperopia, 46 years for emmetropia and 37 years for myopia and there was a statistical significance difference (all *p* < 0.001). The median value of SE was 0.5 D for hyperopia, 0 D for emmetropia and − 1.25 D for myopia and there was a statistical significance difference (all *p* < 0.001). A higher value of divergence was found in the myopic group than in the emmetropic group (*p* = 0.001), but no significant differences were found between the myopic and hyperopic groups (*p* > 0.05) or the hyperopic and emmetropic group (*p* > 0.05). No significant differences were found among the three groups in the value of convergence and FVR (*p* = 0.633 and *p* = 0.177, respectively). Linear regression analysis showed negative correlations between SE and divergence (r = -0.192, *p* = 0.003) (Fig. 1).

**Table 2:** Age, SE and the amplitudes of motor fusion among different refractive groups

	Hyperopia (*n* = 27)	Myopia (*n* = 123)	Emmetropia (*n* = 93)	H	P value
**Age (years)**	54 (49, 56)	37 (31, 45)	46 (40, 51)	58.690	< 0.001*
**SE (D)**	0.5 (0.375, 0.75)	-1.25 (-2.87, -0.75)	0 (-0.25, 0)	177.027	< 0.001*
**Divergence (°)**	9 (8, 10)	10 (8, 12) #	8 (6, 10.5)	11.714	0.003*
**Convergence (°)**	20 (14, 24)	19 (14, 24)	18 (12, 22)	0.913	0.633
**FVR (°)**	30 (24, 33)	30 (22, 36)	28 (20, 33)	4.295	0.117

All data were expressed as Median (1st quartile, 3rd quartile). # Myopia versus Hyperopia, *p* = 0.001. SE: spherical equivalent. FVR: fusional vergence range

**Fig. 2:**
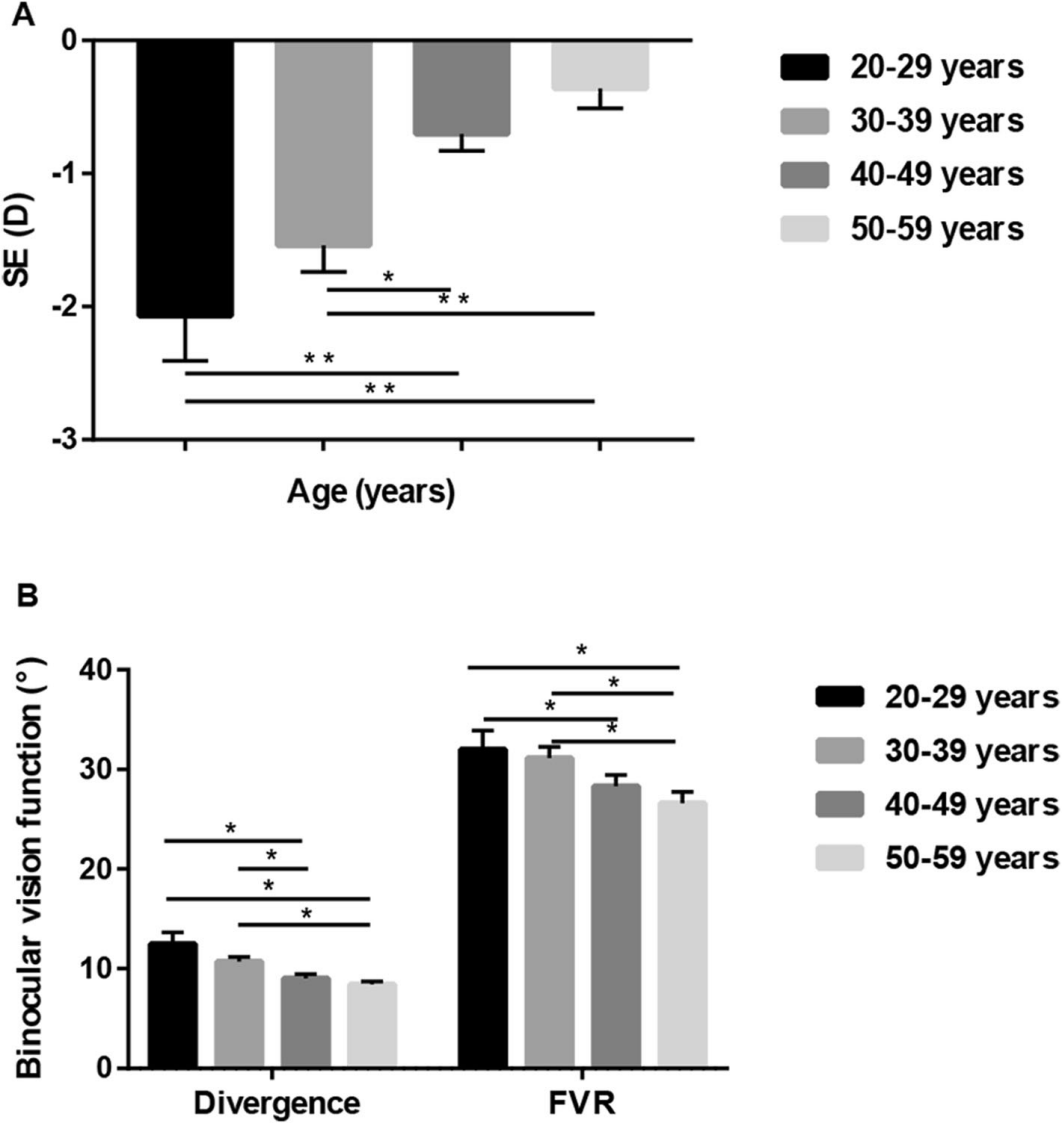
Comparisons of SE and motor fusion among different age groups: (**A**) SE in different age groups, (**B**) divergence and FVR in different age groups. * represent significant differences between two groups (**p < 0.01, *p < 0.05). SE, spherical equivalent. FVR: fusional vergence range

**Fig. 3:**
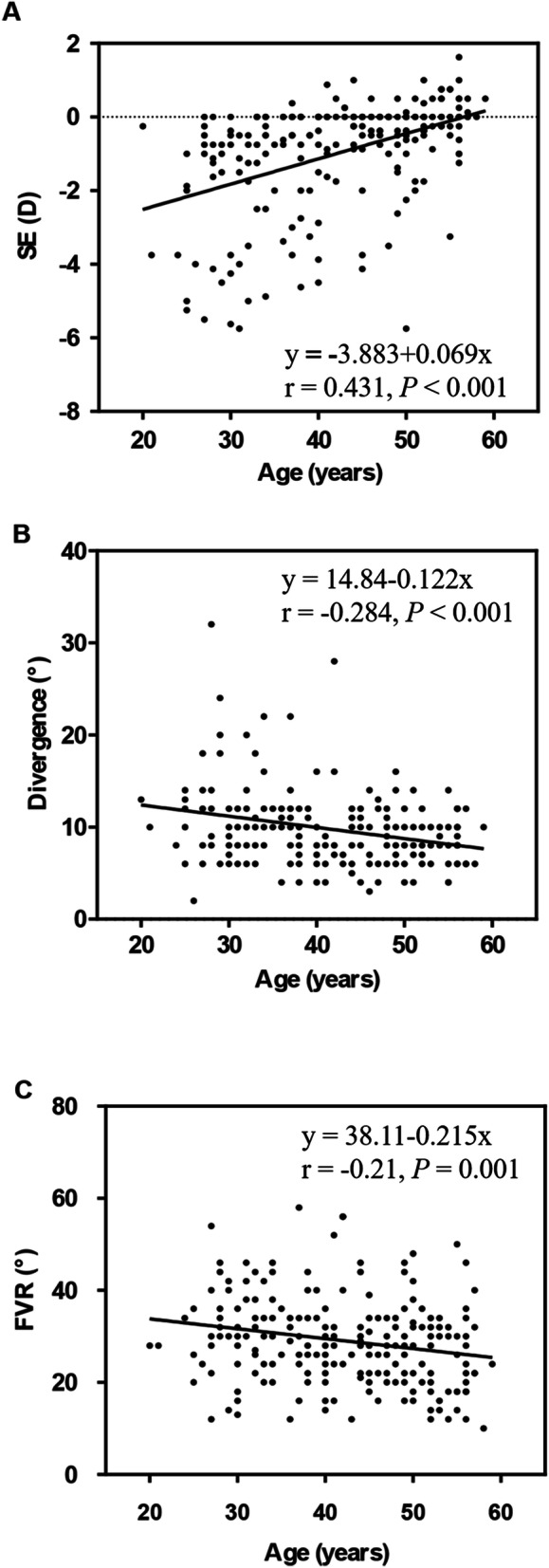
The relationship between age and SE, divergence and FVR, (**A**) SE and age, (**B**) divergence and age, (**D**) FVR and age. The mean age was 42.1 ± 0.61 years, ranging from 20–59 years. SE, spherical equivalent. FVR: fusional vergence range

SE and the amplitudes of motor fusion in different age groups are shown in Table 3. The values of SE, divergence and FVR were significantly different among age groups (*p* < 0.001, *p* < 0.001 and *p* = 0.008, respectively). A post hoc test showed statistically significant differences in SE, divergence and FVR in the 20–29 and 30–39 years age groups compared to the 40–49 and 50–59 years groups (all *p* < 0.05) (Fig. 2). Linear regression analysis revealed that age was associated with SE and divergence. With increasing age, an increasing tendency was observed in SE (r = 0.431, *p* < 0.001; Fig. 3 A), while decreasing tendencies in divergence (r = -0.284, *p* < 0.001; Fig. 3B) and FVR (r = -0.21, *p* = 0.001; Fig. 3 C) were observed.

**Table 3:** SE and the amplitudes of motor fusion among different age groups

	20–29 years (*n* = 27)	30–39 years (*n* = 70)	40–49 years (*n* = 76)		50–59 years (*n* = 70)		H	P value
**SE (D)**	-1.25 (-3.75, -0.75)	-0.75 (-2.5, -0.47)	-0.375 (-0.875, 0)	0 (-0.5,0.156)		47.897		< 0.001*
**Divergence (°)**	12 (8, 14)	10 (8, 12)	8 (6, 11)		8 (6, 10)	23.341		< 0.001*
**Convergence (°)**	20 (14, 22)	19.5 (14, 24.5)	18 (14, 22)		19 (12, 24)	2.081		0.556
**FVR (°)**	32 (26, 40)	31 (24, 38)	28 (22, 32)		28 (18, 32)	11.767		0.008*

All data were expressed as Median (1st quartile, 3rd quartile). SE: spherical equivalent. FVR: fusional vergence range

The proportion of normal SA was 25.9 % in the hyperopic group, 47.3 % in the emmetropic group, and 50.4 % in the myopic group. The proportion of abnormal SA was 74.1 % in the hyperopic group, 52.7 % in the emmetropic group, and 49.6 % in the myopic group. There were no significant differences in the proportion of normal SA among refractive groups (χ2 = 5.373, *p* = 0.068). As shown in Table 4, the proportions of normal SA in the 20–29 years group, 30–39 years group, 40–49 years group and 50–59 years group were 63.0 %, 52.9 %, 31.3 %, and 34.3 %, respectively. The proportions of abnormal SA in the 20–29 years group, 30–39 years group, 40–49 years group and 50–59 years group were 37.0 %, 47.1 %, 53.9 %, and 65.7 %, respectively. There were significant differences in the proportions of normal SA among age groups (χ2 = 8.283, *p* = 0.041).

**Table 4:** The proportion of normal SA (The values of SA satisfied Percival’s criterion)

		20–29 years		30–39 years	40–49 years			50–59 years		Total
**Normal**	17 (63.0 %)		37(52.9 %)			35(31.3 %)	24 (34.3 %)			111
**Abnormal**	10 (37.0 %)		33(47.1 %)			41(53.9 %)	46 (65.7 %)			132
**Total**	27		70			76	70			243

SA: subjective angle. χ2 = 8.283, *p* = 0.041

## Discussion

In the present study, the amplitude of motor fusion, including divergence and convergence, in a Chinese normal adult population with different ages and refractive statuses was reported. For the whole sample group, the average divergence amplitude was 9.72 ± 0.26°, and the mean convergence amplitude was 19.34 ± 0.54°, which agrees with previous studies [6, 20]. Ohyagi et al. [20]reported that the normal divergence was 6° to 10° and the normal convergence was 15° to 20°. Vidhyapriya et al. [6] reported that the divergence range was 15.2 ± 5.9 PD and the convergence range was 38.4 ± 2.1 PD in a small sample of expert adults (*n* = 5) using a lightweight prism bar apparatus, which is similar to our results, as 1 degree of arc is approximately equal to 2 PD [3]. However, in a population of young female athletes (average aged 21.55 ± 0.67 years), the mean values of divergence and convergence were 4.74 ± 1.93 ° and 12.38 ± 8.20°, which was approximately half of the values in this study [3]. This may be due to different instruments used. In addition, different demographic characteristics of the subjects (ages, refractive status) may also account for these discrepancies.

In this study, myopic subjects had larger divergence than emmetropic subjects. In addition, the divergence values were correlated with SE values. These results were in accordance with the previous study. Though the comparison among different refractive groups were not analysed in Ma et al.’s [10] study, a slightly increase of the divergence values in the myopes compared to the emmetropes was shown in their study as they reported the mean values of distance negative fusional vergence break point in emmetropes, low myopes, moderate myopes and high myopes were 11.7 PD, 12.7 PD, 12.8 PD and 12.6 PD, respectively. In a population of university students, no significant difference was found in fusion amplitude in myopes compared to hyperopes [15]. In agreement with that, no significant difference in the convergence amplitude and FVR was observed among the different refractive groups in this study.

Linear regression analysis showed that divergence amplitude and FVR decreased with age. Comparisons of divergence and FVR among different age groups showed statistically significant differences in groups of 20–29 and 30–39 years when compared to 40–49 and 50–59 years, suggesting that divergence and FVR had age-related differences (such as under 40 years and over 40 years). However, the results of some previous studies reported no effect of age on FVR [5, 7]. One possible explanation is the different age ranges used, as the subjects enrolled in these studies were limited to older children (6–12 years), without adult controls. Although the near fusion range was not tested in this study, Vesely’s [21] study reported a positive correlation between the far and near fusion ranges. Therefore, the decreased FVR measured in distant areas may somehow predict a decreased tendency of the near fusion range. It has been known for over a century that the accommodative system declines with age, and heterophoria and fixation disparities increase with age [22, 23]. From as early as childhood, the subjective amplitude of accommodation declines with age in response to ciliary muscle contraction, increased lens thickness, decreased lens diameter and increased lens curvature [24]. These factors may account for the decreases in divergence and FVR. Furthermore, with the increasing of age, an alteration may be found in the relationship between ciliary muscle contraction and accommodative response, and in adaptability of tonic accommodation and vergence [25]. Bruce’s [25] study have confirmed an increase in the accommodative-convergence/accommodation (AC/A) ratio and a decrease in the convergence-accommodation/convergence (CA/C) ratio with age. Similar results were found in Rosenfield’s study [26]. These results indicate an alteration in accommodation-vergence interactions with age, which may cause changes of the amplitudes of fusional vergence. However, no correlation was shown between age and convergence in this study, which was consistent with the work of Jiang et al [27]. Interestingly, Provines’s [28] study found a significant decrease in the amplitude of convergence with increasing age. This may be due to the different vision status of the subjects. In this study, all subjects with refractive errors were told to wear the glasses that provided BCVA when attending the synoptophore exam to measure the far fusional vergences, while spectacles were not worn for the subjects and the amplitudes of convergence were measured for near in Provines’s study.

With increased age, the interval between 35 and 44 years has been described as the early phase of presbyopia [29]. Patients in this stage were more likely to complain about eyestrain or asthenopia among those with longer durations of near work. Moreover, age-related changes in the ciliary muscle of human eyes may also be related to the occurrence of asthenopia. It was reported that the total area and length of the ciliary muscle and the distance of the inner apex of the muscle to the scleral spur showed a continuous and significant decrease with age. This means that the ciliary muscle adopts an anterior-inward position with increasing age, which is a similar form seen in young eyes after ciliary muscle contraction [30]. In a clinical study, subjects with asthenopia showed a lower tendency of relaxation of ciliary muscle when looking at a distant object than subjects without asthenopia [31]. Therefore, the occurrence of asthenopia may be caused by strain of the ciliary muscles even if the patient was looking at a distant target and may occur more frequently in older patients. In this study, the proportion of normal SA (satisfied Percival’s criterion) had age-related differences. The results showed that the proportion of patient with abnormal SA was ranked as 50–59 years > 40–49 years > 30–39 years > 20–29 years. This result indicates that the symptoms of asthenopia may correlate with abnormal SA and that it may be useful to measure synoptophore parameters to predict asthenopia in eyestrain patients. However, further studies are warranted to confirm the association of normal SA in the comfort zone and the occurrence of asthenopia. On the other hand, although the highest proportion of abnormal SA was observed in the hyperopic group, the difference was not statistically significant. In a population of university students with a mean age of 22.8 ± 3.1 years, the prevalence of asthenopia was higher in hyperopic students and astigmatic participants based on cycloplegic refraction [32]. However, in a population of video display operators with a mean age of 46.5 ± 9.3 years, no significant association was found between visual fatigue and refractory disorders with the classification based on lenses in use using a lensmeter [33]. Therefore, this discrepancy may be due to different ages, classifications of refractive status, and different criteria of asthenopia.

There may be several limitations in this study. First, this study had a small sample size, and the ages ranged from 20 years to 59 years; thus, further studies with larger samples and subjects, including juveniles under 20 years and elderly people over 60 years, are warranted. Moreover, we did not evaluate the asthenopia symptoms of the subjects, which we plan to consider in future studies to investigate the relationship between asthenopia and normal SA.

## Conclusions

In conclusion, the normative parameters of motor fusion measured by synoptophore in a healthy Chinese adult population were shown in this study. Refractive error type was correlated to divergence amplitude. Additionally, age was correlated with refractive error type, divergence and FVR. In addition, abnormal SA, which did not satisfy Percival’s criterion, was associated with age, which may be a way to predict those who are at risk of suffering from dysfunctions of motor fusion. Our results are probably negatively influenced by the small number of subjects and the uneven percentage of the subjects in the male and female groups.

## Data Availability

Data are available in a public, open access repository. Data are available upon reasonable request.

## Abbreviations

SE: Spherical equivalent
SA: subjective angle
FVR: fusional vergence range
VA: visual acuity
BCVA: best corrected visual acuity

## References

[1] Pateras E, Tzamouranis D (2020). Technique for measuring strabismus with synoptophore-review. Asian Journal of Research Reports in Ophthalmology.

[2] Laby DM, Kirschen DG, Pantall P (2011). The visual function of olympic-level athletes-an initial report. Eye Contact Lens.

[3] Zwierko T, Puchalska-Niedbał L, Krzepota J, Markiewicz M, Woźniak J, Lubiński W (2015). The Effects of Sports Vision Training on Binocular Vision Function in Female University Athletes. Journal of Human Kinetics.

[4] Aslin RN, Dumais ST (1980). Binocular vision in infants: a review and a theoretical framework. Advances in Child Development and Behavior.

[5] Han D, Jiang D, Zhang J, Pei T, Zhao Q (2018). Clinical study of the effect of refractive status on stereopsis in children with intermittent exotropia. BMC Ophthalmology.

[6] Sreenivasan V, Babinsky EE, Wu Y, Candy TR. Objective measurement of fusional vergence ranges and heterophoria in Infants and preschool children. Investigative Opthalmology Visual Science. 2016;57(6):2678–88. 10.1167/iovs.15-17877 PMC4874477 27183054

[7] Vesely P, Synek S (2013). Simple binocular vision examination on synoptophore determination of normative database of healthy adult subjects examination of binocular vision on synoptophore. Collegium Antropologicum.

[8] Fu T, Wang J, Levin M, Su Q, Li D, Li J (2015). Fusional vergence detected by prism bar and synoptophore in chinese childhood intermittent exotropia. Journal of Ophthalmology.

[9] Dwyer P, Wick B (1995). The influence of refractive correction upon disorders of vergence and accommodation. Optometry Vision Science: Official Publication of the American Academy of Optometry.

[10] Ma MM, Yeo ACH, Scheiman M, Chen X (2019). Vergence and accommodative dysfunctions in emmetropic and myopic Chinese young adults. Journal of Ophthalmology.

[11] Ma MM, Long W, She Z, Li W, Chen X, Xie L, Scheiman M, Liu Y, Chen X (2019). Convergence insufficiency in Chinese high school students. Clinical Experimental Optometry.

[12] Nunes AF, Monteiro PML, Ferreira FBP, Nunes AS (2019). Convergence insufficiency and accommodative insufficiency in children. BMC Ophthalmology.

[13] McGregor ML. Convergence insufficiency and vision therapy. Pediatric Clinics of North America. 2014;61(3):621–30. 10.1016/j.pcl.2014.03.010 24852157

[14] O’Connor AR, Birch EE, Anderson S, Draper H (2010). Relationship between binocular vision, visual acuity, and fine motor skills. Optometry and Vision Science..

[15] Risovic DJ, Misailovic KR, Eric-Marinkovic JM, Kosanovic-Jakovic NG, Milenkovic SM, Petrovic LZ. Refractive errors and binocular dysfunctions in a population of university students. European Journal of Ophthalmology. 2008;18(1):1–6. 10.1177/112067210801800101 18203077

[16] Shibata T, Kim J, Hoffman DM, Banks MS (2011). The zone of comfort: Predicting visual discomfort with stereo displays. Journal of Vision.

[17] Percival AS (1892). The relation of convergence to accommodation and its practical bearing. Ophthalmological Review.

[18] Xu D, Lu F, Qu J (1999). Sheard and Percival criteria inbinocular vision. Chinese Journal of Optometry Ophthalmology Visual Science.

[19] Flitcroft DI, He M, Jonas JB, Jong M, Naidoo K, Ohno-Matsui K, Rahi J, Resnikoff S, Vitale S, Yannuzzi L (2019). IMI – defining and classifying myopia: a proposed set of standards for clinical and epidemiologic studies. Investigative Opthalmology Visual Science.

[20] Ohyagi Y, Yamada T, Okayama A, Sakae N, Yamasaki T, Ohshima T, Sakamoto T, Fujii N, Kira J. Vergence disorders in patients with spinocerebellar ataxia 3/Machado-Joseph disease: a synoptophore study. Journal of the Neurological Sciences. 2000;173(2):120–3. 10.1016/s0022-510x(99)00309-3 10675655

[21] Sreenivasan V, Babinsky EE, Wu Y, Candy TR. Objective measurement of fusional vergence ranges and heterophoria in infants and preschool children. Investigative Opthalmology Visual Science. 2016;57(6):2678–88. 10.1167/iovs.15-17877 PMC4874477 27183054

[22] Duane A. An attempt to determine the normal range of accommodation at various ages, being a revision of Donder’s experiments. Transactions of the American Ophthalmological Society. 1908;11(Pt 3):634–41. PMC1322369 16692147

[23] Yekta AA, Pickwell LD, Jenkins TC. Binocular vision, age and symptoms. Ophthalmic and Physiological Optics. 1989;9(2):115–20. 10.1111/j.1475-1313.1989.tb00829.x 2622645

[24] Davies LN, Croft MA, Papas E, Charman WN. Presbyopia: physiology, prevention and pathways to correction. Ophthalmic and Physiological Optics. 2016;36(1):1–4. 10.1111/opo.12272 26769325

[25] Bruce AS, Atchison DA, Bhoola H. Accommodation-convergence relationships and age. Investigative Ophthalmology Visual Science. 1995;36(2):406–13. 7843910

[26] Rosenfield M, Ciuffreda K, Chen H. Effect of age on the interaction between the AC/A and CA/C ratios. Ophthalmic and Physiological Optics. 1995;15(5):451–5. 8524573

[27] Jang JU, Park IJ, Jang JY (2016). The distribution of near point of convergence, near horizontal heterophoria, and near vergence among myopic children in South Korea. Taiwan Journal of Ophthalmology.

[28] Provines FW. The effects of aging on the amplitude of convergence. Optometry and Vision Science. 1971;48(6):479–83. 5281066

[29] Pointer JS, Gilmartin B. Patterns of refractive change in myopic subjects during the incipient phase of presbyopia: a preliminary study. Ophthalmic and Physiological Optics. 2011;31(5):487–93. 10.1111/j.1475-1313.2011.00832.x 21410500

[30] Tamm S, Tamm E, Rohen JW (1992). Age-related changes of the human ciliary muscle. A quantitative morphometric study. Mechanisms of Ageing Derelopment.

[31] Masayoshi Kajita MO (2001). Setsuko Suzuki, Keiichiro Kato: **Accommodative Microfluctuation in Asthenopia Caused By Accommodative Spasm**. Fukushima Journal of Medical Sciences.

[32] Hashemi H, Saatchi M, Yekta A, Ali B, Ostadimoghaddam H, Nabovati P, Aghamirsalim M, Khabazkhoob M (2019). High prevalence of asthenopia among a population of university students. Journal of Ophthalmic Vision Research.

[33] Larese Filon F, Drusian A, Ronchese F, Negro C (2019). Video display operator complaints: a 10-year follow-up of visual fatigue and refractive disorders. International Journal of Environmental Research Public Health.

